# Microbe-host interactions: structure and functions of Gram-negative bacterial membrane vesicles

**DOI:** 10.3389/fmicb.2023.1225513

**Published:** 2023-08-31

**Authors:** Min Xiao, Guiding Li, Hefeng Yang

**Affiliations:** ^1^Yunnan Key Laboratory of Stomatology, Kunming Medical University, Kunming, Yunnan, China; ^2^Department of Dental Research, The Affiliated Stomatology Hospital of Kunming Medical University, Kunming, Yunnan, China

**Keywords:** Gram-negative, membrane vesicles, interactions, immune response, application

## Abstract

Bacteria-host interaction is a common, relevant, and intriguing biological phenomena. The host reacts actively or passively to the bacteria themselves, their products, debris, and so on, through various defense systems containing the immune system, the bacteria communicate with the local or distal tissues of the host via their own surface antigens, secreted products, nucleic acids, etc., resulting in relationships of attack and defense, adaptation, symbiosis, and even collaboration. The significance of bacterial membrane vesicles (MVs) as a powerful vehicle for the crosstalk mechanism between the two is growing. In the recent decade, the emergence of MVs in microbial interactions and a variety of bacterial infections, with multiple adhesions to host tissues, cell invasion and evasion of host defense mechanisms, have brought MVs to the forefront of bacterial pathogenesis research. Whereas MVs are a complex combination of molecules not yet fully understood, research into its effects, targeting and pathogenic components will advance its understanding and utilization. This review will summarize structural, extraction and penetration information on several classes of MVs and emphasize the role of MVs in transport and immune response activation. Finally, the potential of MVs as a therapeutic method will be highlighted, as will future research prospects.

## Introduction

It is becoming increasingly clear that the microbiota has a profound influence on multiple aspects of host development and physiology, including metabolism, nutrient acquisition, and the immune system (Sommer and Backhed, 2013). They are linked to the susceptibility and the progression of diseases such as diabetes, obesity, inflammatory bowel disease, and cancer in humans (Choi et al., 2018; Tan et al., 2019; Albillos et al., 2020). The complex symbiotic and coevolutionary processes between host and microorganisms have established strong links between microbes and host phenotypes (Li et al., 2019), as well as disease development. The mechanisms of bidirectional communication between microbes and hosts, as well as microbe-microbe interactions, are still unknown.

Microorganisms can significantly affect host traits and diseases by influencing metabolism and other processes that regulate host phenotype, organismal immunity, and disease (Tan et al., 2019; Albillos et al., 2020). Some researchers argue that host–microbe interactions are primarily environment dependent (Haller and Autenrieth, 2010), and that the immune system tolerates beneficial commensal bacteria during immune homeostasis. However, if tissue destruction or other homeostatic perturbations, the tissue would be injured by the same microbiota responses (Gensollen et al., 2016). We should start with the symbiotic function and integrate different approaches to study the interaction mechanism between microbiota and hosts (Rosenberg and Zilber-Rosenberg, 2018). Bacteria have developed numerous mechanisms for adhering and invading host cells. Bacteria can thus enter the bloodstream and spread throughout the body, affecting organ function. It has been suggested that bacteria and their products, rather than bacteria themselves, activate the body’s monocytes/macrophages and produce a large number of inflammatory factors (Agarkov et al., 2020), including endotoxin, LPS, pore proteins, and polysaccharides. In addition to this, the membrane vesicles (MVs) discovered in recent decade seem to have the similar effect. What’s exciting for us is that MVs are particularly important in transport, virulence, inflammation, and immune interactions with host cells. Pathogen-associated molecular patterns (PAMPs) can be transported to immune cells by MVs, which can then be activated intracellularly to transcribe and translate related proteins and interact with host cell membranes as transporter proteins (Furuyama and Sircili, 2021; Gilmore et al., 2021). MVs can also function as biomolecular carriers, mediate host cell endocytosis and biosignaling, and serve as drug delivery systems or delivery vehicles. Previously, MVs research was primarily focused on microbial infection and transmission mechanisms. The lack of a thorough understanding of their interactions with the host will limit their use in biomedical fields. As a result, the article starts with the types and formation mechanisms of membrane vesicles from bacteria, focuses on the highlights of MV-host cell interactions such as transport, virulence, inflammation, immunity, and others. Furthermore, it introduces clinical applications of MVs to provide theoretical support for MVs biomedical and clinical applications. Finally, some novel ideas for MVs research and application have been proposed. A more extensive explanation follows.

## Types, formations, and extraction of MVs

### Types and formations of MVs

Bacterial vesicles were initially found to arise through controlled blistering of the outer membrane of Gram-negative bacteria and are therefore commonly referred to as outer membrane vesicles (OMVs). However, as the study of vesicles became more advanced, one type of the vesicles produced by Gram-negative bacteria was named OMVs, although in most cases, the term “outer membrane vesicles” (OMVs) is used specifically to refer to vesicles formed by Gram-negative bacteria. To avoid misunderstanding, we use MVs stands for OMVs from Gram-negative bacteria. MVs generation and secretion is a complicated and finely tuned process and has been thoroughly summarized in the literature. Moreover, MVs are highly heterogeneous in nature, and the biophysical and biochemical properties of MVs often depend on the type. It is important to understand the MVs types and their individual features before exploring their functional properties. Gram-negative bacteria may create vesicles, which include outer and inner membrane vesicles (OIMVs), cytoplasmic membrane vesicles (CMVs), and tube-shaped membranous structures (TSMSs), in addition to outer membrane vesicles (OMVs). Various formation paths result in different vesicle models, and their designs reflect their formation routes. Their composition and substance may influence its function. Table 1 shows the specific contents.

**Table 1:** Contents contained in MVs.

Vesicle types	Outer membrane proteins	Cytoplasmic (or inner) membrane proteins	Plasmids	RNA and chromosomal DNA	Endolysins	Virulence factors	Hydrophobic molecules	Phages
Outer-membrane vesicle (Gram-negative)	+	-(+)	?	-(+)	−	+	+	−
Explosive outer-membrane vesicle (Gram-negative)	+	+	+	+	+	+	+	+
Outer-inner membrane vesicle (Gram-negative)	+	+	+	+	+	+	+	+
Cytoplasmic membrane vesicle (Gram-positive)	NA	+	+	+	+	+	−	+
Tube-shaped membranous structure (Gram-negative)	+	−	−	−	?	−	−	−
Tube-shaped membranous structure (Gram-positive)	NA	−	+	+	?	?	−	−

+, present; −, absent;? unknown; NA, not applicable.

#### Outer membrane vesicles

**Figure 1:**
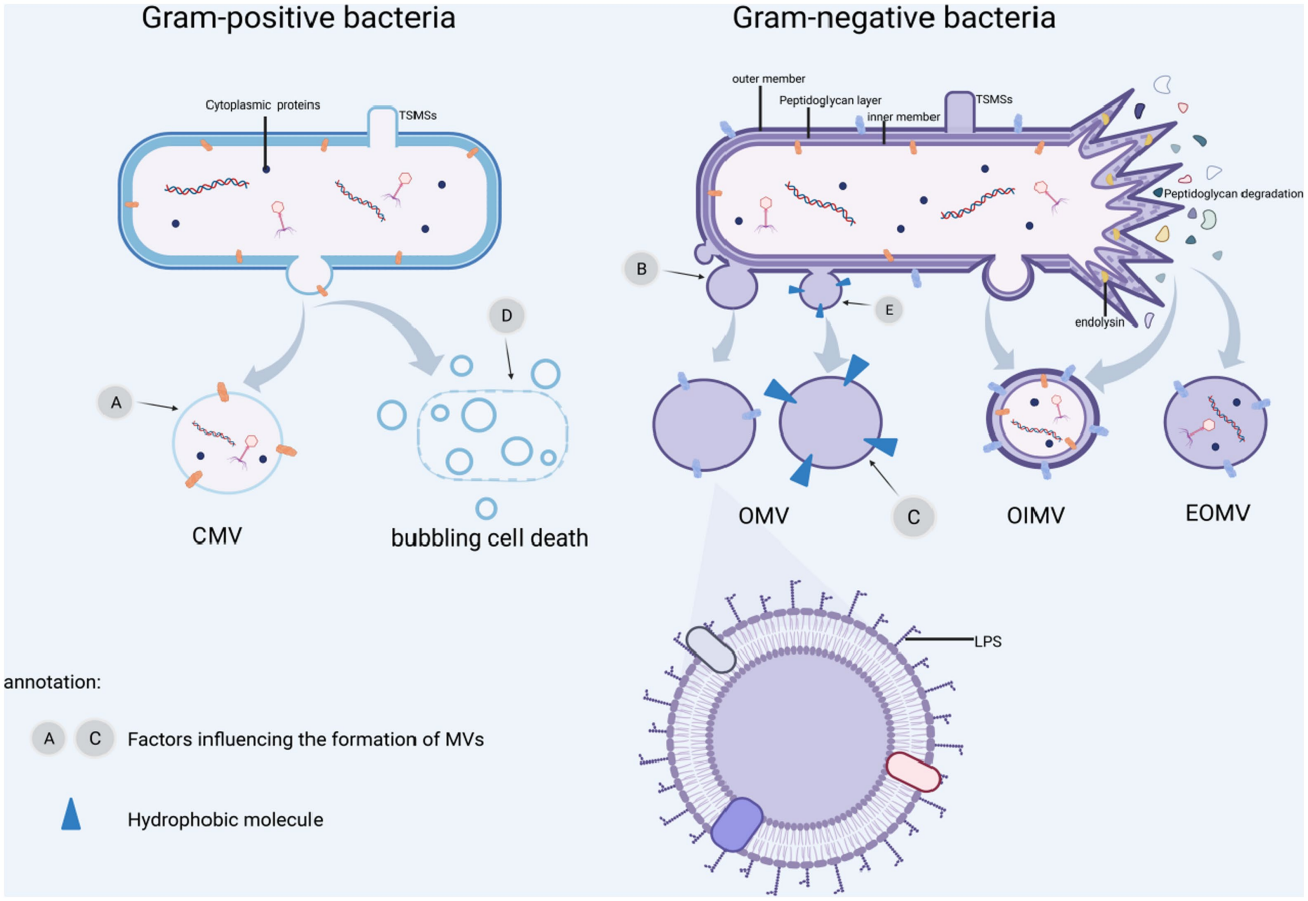
MVs formation and influence factors. **(A)** *β*-lactamase affects cell wall biosynthesis and ciprofloxacin causes DNA damage. **(B)** Biosynthetic imbalance, misfolded proteins buildup, leading to outer membrane protrusion. **(C)** Outer membrane stress is caused by abnormal iron-restricted phosphate transport, gentamicin and mucin. **(D)** Induction of SOS response by DNA damaging agents, UV rays, ciprofloxacin and other external circumstances resulting in “bubbling cell death.” **(E)** Insertion of hydrophobic molecules such as PQS and toluene, leading to altered outer membrane curvature. BioRender.com was used to create this.

Outer membrane vesicles (OMVs) are vesicle-like spherical structures formed spontaneously by Gram-negative bacteria (Bitto and Kaparakis-Liaskos, 2017; Toyofuku et al., 2019) during normal growth (Schwechheimer and Kuehn, 2015). OMVs secretion is a strain-specific and selective process (Jan, 2017). OMVs generated from the Gram-negative bacterial outer membranes are spherical lipid bilayer nanostructures range in size from 20 to 300 nm (Jan, 2017). As illustrated in Figure 1, they comprise a range of parent bacterial-derived components (Li et al., 2020), which are mostly enzymes, bacterial-specific antigens, virulence factors, and PAMPs such as LPS, peptidoglycan, lipoproteins, bacterial DNA, RNA (Kaparakis-Liaskos and Ferrero, 2015). The outermost layer of OMVs includes the majority of the pathogenic compounds found in Gram-negative bacteria, and it is composed of three layers: lipid A, core polysaccharide layer, and O-antigen (Hao et al., 2015). OMVs from Gram-negative bacteria have lipopolysaccharide and outer membrane proteins incorporated in their membranes (Bitto and Kaparakis-Liaskos, 2017).

OMVs are often formed by blebbing of the outer membrane (Kulp et al., 2015; Orench-Rivera and Kuehn, 2016; Roier et al., 2016), in which the cell wall component, peptidoglycan production, is disrupted or hydrophobic molecules are injected into the outer membrane, damaging the cell envelope, and OMVs protrude from the outer membrane area. Disruption of the crosslink between the peptidoglycan and the outer membrane, resulting in their separation. Moreover, the buildup of peptidoglycan fragments or misfolded proteins has been observed in the literature, resulting in bulking pressure on the outer membrane and, eventually, OMVs freedom (Henriquez and Falciani, 2023). The population-sensing quinolone signaling (PQS) molecules induce OMVs production by inserting into the outer membrane and mediating its packing and transport, and when PQS in the outer leaflets is cumulative thought to change curvature (Florez et al., 2017). Insertion of hexadecane, 1-phenylbutadiene, and toluene into the outer membrane’s hydrophobic region causes changes in membrane curvature and stimulation of OMVs production (Shetty and Hickey, 2014). Because the inner membrane is still intact, cytoplasmic components cannot come into direct contact with these OMVs (See Figure 1B; Table 1). Gentamicin and mucin also promote OMVs formation (Fulsundar et al., 2014). Hyper vesiculation was caused by deacylation of lipid A and concomitant outer membrane remodeling in *Salmonella typhimurium*, as well as accumulation of phospholipids in the outer leaflet of the outer membrane of *Haemophilus influenzae* and *Vibrio cholera* (Elhenawy et al., 2016; Roier et al., 2016). Iron restriction can regulate OMVs formation in *Escherichia coli*, *V*. *cholera* and *H. influenzae* by affecting the expression of phospholipid transport genes (Roier et al., 2016).

#### Outer and inner membrane vesicles

Outer and inner membrane vesicles (OIMVs) can be produced by explosive cell lysis and blebbing. Endolysin is commonly utilized by ds-DNA phages to split their bacterial hosts, and DNA damage stress induces endolysin expression, resulting in peptidoglycan layer degradation (Turnbull et al., 2016). In contrast to OMVs, peptidoglycan is degraded, cells explode, and membrane fragments aggregate and self-assemble into outer and inner membrane vesicles (OIMVs) and explosive outer membrane vesicles (EOMVs) (See Table 1). Meanwhile, CMVs are released because of phage-triggered cell lysis (Tzipilevich et al., 2017). Under hypoxic condition, explosive cell lysis can be induced (Toyofuku et al., 2014). Endolysin weakens the bacterial peptidoglycan layer. The inner membrane extrudes into the peripheral cytoplasm, allowing inner cytoplasmic matters like DNA entering the vesicles, which ultimately extrude from the bacterial inner cytoplasmic to extracellular space with the surrounding outer membrane. The bacteria produce both EOMVs and OIMVs, but the OIMVs contain more DNA (see Figure 1). The SOS reaction causes phage-encoded endolysins to be expressed to accelerate vesicle formation, and this pathway appears to be the predominant route of OIMV production (Bauwens et al., 2017). Exposure to DNA-damaging chemicals or UV radiation, as well as quinolones such as ciprofloxacin, might trigger the SOS response (Bernier et al., 2013).

#### Cytoplasmic membrane vesicles

Gram-positive bacteria *Bacillus subtilis* are capable of releasing cytoplasmic membrane vesicles containing PBSX phage particles. PBSX-encoded endolysins (Toyofuku et al., 2017) or cell wall-weakening antibiotics such as *β*-lactam (Biagini et al., 2015) lead a peptidoglycan layer to form a pore through which the cytoplasmic membrane protrudes to produce CMVs. Endolysins in thick-walled Gram-positive bacteria promote partial hydrolysis of the bacterial cell wall, a condition known as “bubbling cell death.” CMVs formation in surrounding bacteria are triggered by endolysin released after death (Toyofuku et al., 2017; see Figure 1D).

#### Tube-shaped membranous structure

This is a tubular protrusion of the outer membrane of Gram-negative bacteria or a localized cleavage of the Gram-positive cell wall resulting in a protrusion of the cytoplasmic membrane (Dubey et al., 2016), which frequently decorates on the surface of the resulting cells as well connects with cells to allow the exchange of diverse cellular components (Baidya et al., 2018). This tubular structure has received less attention, been less studied, and the precise method of its development is still unknown.

Bacterial extracellular vesicles are somewhat different from extracellular vesicles (EVs). EVs are tiny biological nano-vesicles, approximately 30–3,000 nm in diameter, released into most extracellular matrix and biological fluids (Théry et al., 2018). Among the classifications of vesicles, the most common are exosomes, microvesicles, and apoptotic vesicles (Gavard, 2023). EVs contain parental cell-derived proteins, mRNA, miRNA, lipids, and small molecule metabolites, etc., which can be transported to the recipient cells for action, mediating intercellular communication (van Niel et al., 2022). Thus, EVs regulate various critical physiological and pathological processes, such as immune regulation. They can be used as disease diagnostic markers, therapeutic agents, drug targets, and drug carriers (Zhu et al., 2017; Xu et al., 2018). EVs are essential in antimicrobial defense, allergy, autoimmune, and antitumor immune responses, contributing to organ development and progress in cancer therapy.

### Extraction method of MVs

Despite the fact that MVs have diverse biological roles and clinical application potential, there are still challenges in bacteria-host interactions and clinical transformation due to a lack of standard extraction protocols. A summary of existing MVs extraction methods is a crucial starting point for its research. Ultracentrifugation, density gradient centrifugation, ultrafiltration, size-exclusion chromatography, and hydrophilic polymer precipitation are the primary methods for the extracting MVs. Each approach cannot extract the vesicles with perfect morphology, and each has advantages and shortcomings, which are listed in Table 2. To select the best extraction procedure, we must consider the downstream experimental requirements and experimental conditions.

**Table 2:** Advantages and drawbacks of several methods for extracting MVs.

Name	Time consumption	Advantages	Drawbacks	Ref
Ultracentrifugation	140–600 min	Low cost, few reagents and consumables, high volume of specimens, no additional chemicals.	High equipment requirements, operational complexity and time, contamination of the finished product (presence of protein aggregates, apoptotic vesicles and other particles), low RNA yields, vesicles can be damaged; efficiency is influenced by rotor type, force magnitude, and sample viscosity.	Momen-Heravi et al. (2013), Livshits et al. (2015), Bryzgunova et al. (2016) and Patel et al. (2019)
Density gradient ultracentrifugation	250 min-2 day	Pure formulation; no viral particle contamination after centrifugation of iododiol; no additional chemicals.	Small capacity, complex, laborious and time-consuming, expensive equipment, samples can be lost, contamination of viral particles in sucrose density gradient method, long procedure time, low yield.	Van Deun et al. (2014), Greening et al. (2015), Lobb et al. (2015) and Abramowicz et al. (2016)
Ultrafiltration	130 min	Simple procedure, allowing simultaneous processing of many samples; pure formulations; additional chemicals; no limitations on sample volume.	Filter clogging, sample loss (large size vesicle rupture), protein contamination, vesicle deformation, small amounts of exosomal proteins.	Salih et al. (2014), Lobb et al. (2015) and Taylor and Shah (2015)
Size-exclusive Chromatography	1 mL/min	High purity; maintains vesicle integrity; high sensitivity with no loss; prevents MV aggregation; no additional chemicals.	Specialized equipment; complex operation; no more than one sample processed in each process; high cost.	Boing et al. (2014), Lobb et al. (2015), Nordin et al. (2015) and Taylor and Shah (2015)
Hydrophilic polymer precipitation	65 min	Simple cost and process; maintains MV integrity; no additional equipment required.	Contamination of polymers, potential for co-precipitation of other non-vesicular contaminants.	Andreu et al. (2016) and Gamez-Valero et al. (2016)

#### Ultracentrifugation

For membrane vesicle separation, Ultracentrifugation (UC) has been deemed the method of choice (Momen-Heravi et al., 2013). It works by applying centrifugal force to a solution of macromolecules and exploiting the buoyancy of the particles to deposit them in the order of their density. As the speed of centrifugal force increases, apoptotic vesicles, and cellular debris, as well as loosened vesicles, pellet one after another. Centrifugation at 300–400 × *g* for 10 min allows for cells precipitation, cell debris removal at 2,000 × *g*, and removal biopolymers and apoptotic vesicles at 10,000 × *g* (Lobb et al., 2015). The vesicles in the supernatant are precipitated by ultracentrifugation at 100,000-200,000 × *g* for 2 h (Xu et al., 2016). The effectiveness of centrifugal separation affected by several factors, including gravity acceleration (g), rotor settling angle, rotation radius, pelleting efficiency (rotor and tube k-factor), and viscosity of the separation fluid (Livshits et al., 2015; Abramowicz et al., 2016). As a result, when using and correcting the ultracentrifugation protocol to obtain less mix other impurities vesicles, these influencing factors should be considered.

#### Density gradient ultracentrifugation

To increase the intensity of particle separation, Density gradient ultracentrifugation (DGUC) is based on buoyant density (Hogan et al., 2014). This technique can separate subcellular components like mitochondria, peroxisomes, and nucleosomes. To form a gradient, density gradient ultracentrifugation uses either continuous density gradients (formed during centrifugation or pre-centrifugation) or stepwise gradients (density increases in a discrete manner) (Webber and Clayton, 2013). However, the final vesicle product may contain significant non-MV protein contamination using this method (See Table 2). Two double sucrose layers containing 1 and 2 mol/L sucrose can be used with DGUC (Raj et al., 2012). MVs are fractionated into the layer containing 1 mol/L sucrose, whereas the layer containing 2 mol/L sucrose contains more giant vesicles. The standard density gradient ultracentrifugation protocol with two sucrose layers produces a higher purity MVs preparation (Lobb et al., 2015; Abramowicz et al., 2016).

#### Ultrafiltration

Based on same-diameter pores and a narrow range of pore size distribution, Ultrafiltration (UF) is used to simplify the separation of specific-size particles. Researchers frequently combine MVs separation with microfiltration or ultrafiltration. Ultrafiltration, in particular, can exist in successive stages of ultracentrifugation (Campoy et al., 2016). Ultrafiltration has been shown to separate MVs preparations with low levels of MVs proteins, such as aquaporins and nephrin, from those with high levels of non-MV proteins, such as albumin and 1-antitrypsin (Kim et al., 2022). Ultrafiltration is a quick, easy method that does not require any expensive equipment (Lobb et al., 2015). However, Alvarez et al. (2012) demonstrated that ultrafiltration produces less MVs and less pure RNA (including microRNA) than ultracentrifugation and PEG precipitation (See Table 2).

#### Size-exclusive chromatography

Size-exclusive chromatography (SEC) employs gentle physical conditions to efficiently separate the gel from a large number of soluble macromolecules present in the biological sample, while preserving vesicle integrity and structure (Taylor and Shah, 2015). SEC gelation separates molecules with different hydrodynamic radii, and can also separate MVs from plasma, urine protein complexes, and lipoproteins (Gamez-Valero et al., 2016). Small hydrodynamic radii components of the sample can pass through the pores, resulting in delayed elution, whereas large hydrodynamic components are prevented from entering the pores. However, in order to obtain MVs samples free of protein and lipoprotein impurities, MVs samples must be pretreated with ultracentrifugation or ultrafiltration. Immunoblot analysis of water channel protein-2, a typical microbubble protein, revealed that the chromatographic method is capable of separating a relatively large fraction when compared to the classical (Balaj et al., 2015).

#### Precipitation polyethylene glycols

Proteins, nucleic acids, viruses, and other small particles have long been precipitated by using precipitation polyethylene glycols (PEGs) of varying molecular weights. This method reduces the solubility of compounds in super hydrophilic polymers like polyethylene glycol solutions. Water-free polymers hold water and push insoluble components out of the solution. Samples containing MVs are typically incubated with a precipitation solution containing a polymer (e.g., PEG 8000) at low temperatures and for a relatively long period of time (overnight), after which the MV-rich precipitate is separated by low-speed centrifugation or filtration (Zeringer et al., 2015). PEG precipitation is a simple and fast method that does not distort MVs, can work in the physiological pH range, and is less dependent on ion concentration (Popović and de Marco, 2018). PEG-separated MVs have particle sizes is comparable to ultracentrifugation, ultrafiltration, and chromatography. At the same time, the number of MVs, specific protein molecules, and RNAs is typically much higher (Taylor and Shah, 2015; Andreu et al., 2016). The main disadvantage of this method is that the sample becomes contaminated with insoluble protein aggregates, viruses, and other particles (Lobb et al., 2015).

#### Protein organic solvent precipitation

The method involves precipitating protein in acetone while retaining hydrophobic vesicles in the supernatant (Gallart-Palau et al., 2016). Supernatants containing MVs fractions were concentrated in a vacuum concentrator after samples were spiked four times the volume of cold acetone (−20°C). Western blot assays have revealed that PROSPR-isolated MVs had higher levels of expression of relevant markers than ultracentrifugation (Gallart-Palau et al., 2015). However, cold acetone causes a decrease in enzyme activity. Some scholars have also used saturated ammonium sulfate to precipitate proteins to extract MVs, and although the yield was not high, the enzyme activity was largely unaffected (Cui et al., 2021).

After describing the formation of MVs and the extraction method, the versatile of MVs will be described next. MVs can mediate microbe-microbe and host–microbe interactions, increasing viability and pathogenicity. Furthermore, they may activate macrophages and neutrophils, inhibit antigen presentation by dendritic cells, disrupt with the regulation of T and B cells, stimulate the secretion of inflammatory factors, and even cause cell death. The transport, virulence, inflammation, and immune interactions of MVs with host cells come next.

## MVs functions in bacteria-host interaction

MVs study has recently focused on their contributions to a wide range of physiological and pathophysiological conditions. Following separation from the bacterial cells, MVs translocate and connect with the surface of recipient cells, delivering cargoes to the cytoplasm and activating downstream signaling pathways cascades. To date, it has been well documented that MVs play an important role in a variety of biological processes such as virulence factors delivery, immunomodulation, inflammation, and so on. This section focuses on recent advances in the field of bacteria-host interactions.

### Transport and adhesion between MVs and cells

#### Transmission role

**Figure 2:**
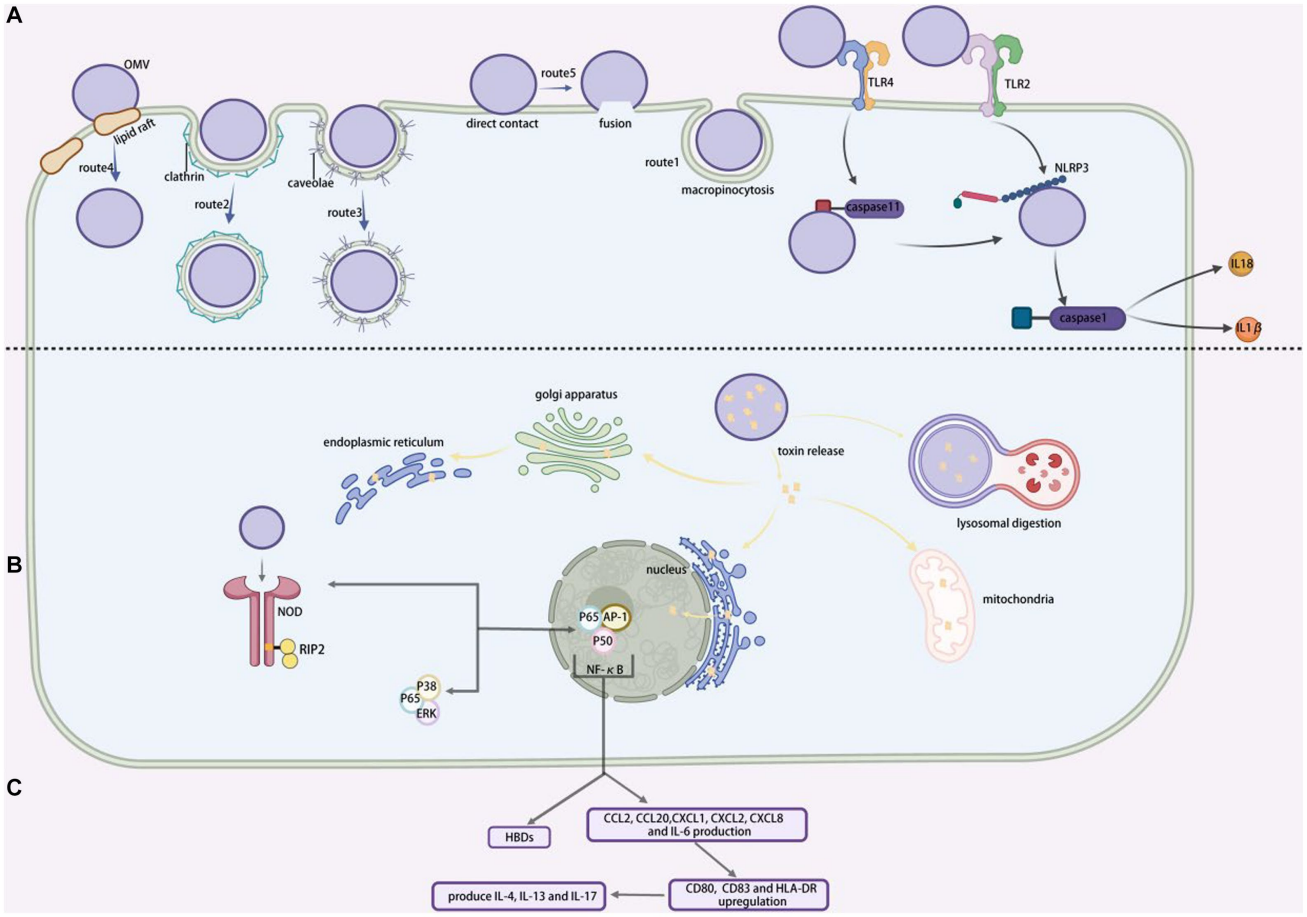
MVs entering into and interacting with cells. **(A)** Various ways for MVs entry into the recipient cells. Route 1, represent macropinocytosis pathway; route 2, represent clathrin-protein-mediated endocytosis pathway; route 3, represent caveolae-mediated endocytosis pathway; route 4, represent lipid raft-mediated endocytosis pathway; route 5, represent direct membrane fusion pathway. **(B)** The toxins released by MVs target particular sites within the host cell. **(C)** NF-*κ*B promotes extracellular production of human beta defensins (HBDs) and increased NOD1-dependent production of CCL2, CCL20, CXCL1, CXCL2, CXCL8 and IL-6, as well as upregulation of CD80, CD83 and HLA-DR, which triggers TH2 and TH17 cellular responses to generate IL-4, IL-13 and IL-17. BioRender.com was used to create this.

MVs are capable of delivering virulence factors and PAMPs. Pathogenic MVs can transfer toxins and PAMPs to host cells, altering host defense mechanism and regulating immunological responses, resulting in infectious diseases (Yoon, 2016). The most prevalent pathogenic factors detected in MVs are virulence factors, which convey a specific virulence factor to specific sections or distal areas of the host cell (As shown in Figure 2B). *Pseudomonas aeruginosa* MVs can transport bioactive compounds such as alkaline phosphatase, phospholipase-C, *β*-lactamase, and Cif into the cytoplasm (Schertzer and Whiteley, 2013). *Pseudomonas aeruginosa* MVs, according to Bomberger et al. (2009), can convey virulence factors to airway epithelial cells. Furthermore, the principal pathogenic toxins of *V. cholera*, including as CT and TCP, can be delivered to host cells in a physiologically active state via MVs (Elluri et al., 2014). Toxins are released by cells during MVs internalization and translocation, stimulating DNA-damaging processes and eventually leading to cell death (Bielaszewska et al., 2018). MVs may transfer lipids and other membrane components between cells simultaneously (Alves et al., 2016), providing them a significant edge in becoming drug carriers. CAGA protein can be transported to cellular endosomes and lysosomes by *H. pylori* outer membrane vesicles can transport via unique absorption processes (Parker and Keenan, 2012).

#### Adhesion and bacterial protection

Bacterial adhesins in MVs compete for binding to host cells (Zhang et al., 2019a). Bacterial adherence to host cells is mediated by MV-carried sticky molecules such as Ata, BabA, SabA, OmpA, and others. MVs then enter the cell as previously stated (see Figure 2A). They bind to pattern recognition receptors (PRRs) once inside the body, activating the immune system and boosting the development of inflammatory reactions. The primary PRRs linked with MVs are Toll-like receptors (TLRs) on the cell surface and NOD-like receptors (NLRs) (Kaparakis-Liaskos and Ferrero, 2015) (see Figure 2A). *Porphyromonas gingivalis* MVs release adhesins (Tanabe et al., 2008), which drive cells to clump and promote biofilm formation in gingival plaque. MVs, for example, promote bacterial adhesion to the intestinal and respiratory epithelia, allowing microorganisms to resist physical clearance (Kim et al., 2013).

Following bacterial invasion, the bacteria will instantly produce massive amounts of MVs, which will encapsulate antibiotics and virulence factors and expel them from the body as a result of temperature fluctuations, oxidative stress, antibiotics, and other conditions. This method successfully eliminates hazardous components in bacteria while maintaining the interconnectedness of the bacterial membrane, hence safeguarding microorganisms (Romanowski et al., 2021). Lysogenic phages break down bacteria by recognizing target receptors on the cell membrane. MVs operate as decoys for lysogenic phages and antibacterial chemicals, providing immediate antibacterial protection (Balhuizen et al., 2021). *Helicobacter pylori* MVs aid pathogen immune evasion by boosting intracellular immunosuppressive cytokines and suppressing the inflammatory responses (Winter et al., 2014). *Porphyromonas gingivalis* MVs contain porphyrin peptide acylcarnitine deiminase (PPAD), which inhibit complement factor C5a function and permit *P. gingivalis* immune evasion (Bielecka et al., 2014).

### Modes of entry of MVs into the host cells

Although there is significant evidence that MVs can penetrate host cells and release their contents, the mechanism by which they are connected with and taken up by host cells are not entirely understood. Turner et al. (2018) hypothesized that the size of MVs might influence their immunogenicity and the process of entry. Smaller MVs preferentially enter non-phagocytic epithelial cells via vesicle protein-dependent endocytosis, whereas large MVs t preferentially endocytosis or macropinocytosis, with larger MVs preferentially entering non-phagocytic epithelial cells via vesicle proteins. Our present understanding of the mechanism involved in MVs uptake will be demonstrated by providing a basic overview of the uptake pathway, followed by a list of example MVs entries. The five endocytic pathways by which MVs may enter non-phagocytosed host cells include macropinocytosis, clathrin-protein-mediated endocytosis, caveolae-mediated endocytosis, lipid raft-mediated endocytosis, and direct membrane fusion (shown by Figure 2).

#### Macropinocytosis

Actin-dependent macropinocytosis is driven by the polymerization of actin rings in the subcellular membrane, which takes the form of a circular fold protrusion that finally closes at the top and encompasses some of the extracellular area (Bloomfield and Kay, 2016). *Shigella fowleri* has been demonstrated to employ this pathway during host cell invasion (Weiner et al., 2016) (shown by Figure 2A, route1).

#### Clathrin-protein-mediated endocytosis

The creation of clathrin-mediated endocytosis is caused by the formation of clathrin-encapsulated pits of up to 200 nm in diameter (shown by Figure 2A, route 2). In contrast to macrophage drinking, ligand attachment to cell surface receptors can trigger internalization (Rewatkar et al., 2015). Many bacterial virulence factors, including Shiga toxin, cholera toxin, and the gingival silver pain adhesin of *Porphyromonas gingivalis*, have been shown to enter host cells via clathrin-protein-mediated endocytosis (Neilands et al., 2019). Another mechanism by which *Haemophilus pylori* MVs can invade human gastric epithelial cells is clathrin-protein-mediated endocytosis (Parker et al., 2010). Although multiple investigations have connected this pathway MVs entry, suitable ligands have yet to be identified. If these interactions are necessary for MVs internalization, then identifying the components involved could lead to the design of inhibitors that minimize infection by preventing the distribution of virulence factors associated with MVs (O’Donoghue and Krachler, 2016).

#### Caveolae-mediated endocytosis

*Pseudomonas aeruginosa*, *Campylobacter jejuni*, *E. coli*, and *S. typhimurium* all use caveolae protein-mediated endocytosis (shown by Figure 2A, route 3)to entry cells (Machado et al., 2012). It is believed that bacteria internalized by vesicles, as opposed to bacteria absorbed by grid protein-coated pits, are able to avoid being transported to lysosomes and subsequently being destroyed (O’Donoghue and Krachler, 2016). This suggests that pathogens may prefer this approach.

#### Lipid raft-mediated endocytosis

Numerous investigations have demonstrated that lipid rafts facilitate MVs penetration (shown by Figure 2A, route 4). Sphingolipid and cholesterol are abundant in the structural regions known as plasma membrane lipid rafts (Mulcahy et al., 2014). The cholesterol-rich regions of the bilayer aggregate and cause the membrane to bend, which leads to host cells invasions and particle entry into the cells (Wang et al., 2022). Early research established that the enterotoxin-producing *E. coli* MVs enter host cells via lipid rafts at different temperatures (Kesty et al., 2004). The MVs of *P. gingivalis*, *H. influenzae*, *P. aeruginosa*, and *C. jejuni* also enter their host epithelial cells using lipid rafts. Another investigation confirmed that *H. pylori* MVs use a cholesterol-dependent method to invade host cells (Olofsson et al., 2014).

#### Direct membrane fusion

Despite structural differences between the bilayers of MVs and host eukaryotic cells, membrane fusion has been described as a method for MVs entry into host cells (shown by Figure 2A, route 5). Using membrane-bound fluorescent dyes like rhodamine R-18, this has been shown to take place preferentially in the lipid raft structural domain in *Legionella pneumophila* (Jager et al., 2015).

### Mediating inflammatory immune responses

Pathogen-associated pattern molecules (PAMPs) such as DNA, RNA, lipoproteins, LPS, peptidoglycan, and others are found in MVs (Zhang et al., 2019b). MVs carry PAMPs that bind PRRs of host immune cells, amplify through signaling cascades, activate inflammatory pathways, disrupt inflammatory factor secretion, contribute to increased synthesis of cellular inflammatory mediators, accumulate inflammatory cells, exacerbate the inflammatory response, and stimulate an MV-specific adaptive immune response (Pathirana and Kaparakis-Liaskos, 2016; Cecil et al., 2017). Additionally, the higher PAMPs level in MVs compared with parental bacteria (Fleetwood et al., 2017) exhibited a substantial pro-inflammatory impact. The differences in host cell responses to MVs and parental bacteria may also be reflected in variation in MVs lipid and protein composition.

**Figure 3:**
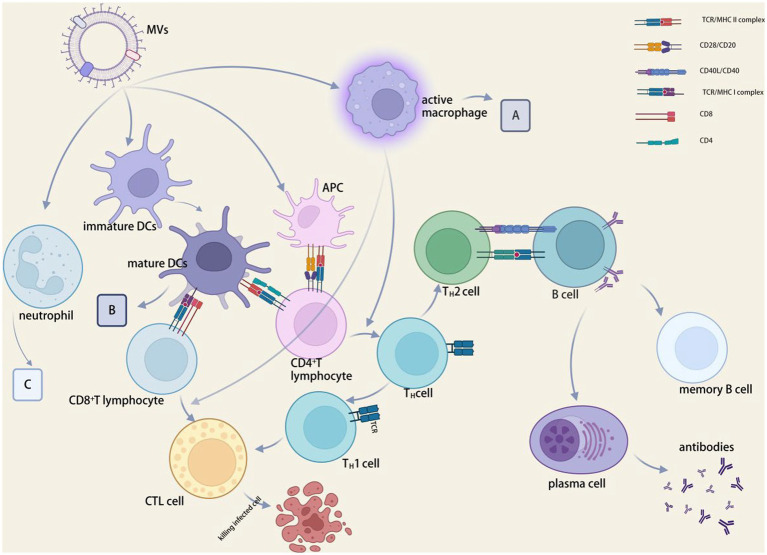
MVs in immunomodulation. **(A)** CD80, CD86, HLA-DR, ICAM1, CCLD2, CCL3, CCL5, CXCL8, IL-1*β*, IL-6, IL-10, IL-12p40, IL-12p70 and TNF expression were increased, and CD14 expression and lipopolysaccharide-mediated responses were decreased. **(B)** CCL2, IFN*γ*, IL-12p40, IL-12p70, IL-16, and TNF levels were elevated, while phagocytic and chemotactic activities were reduced. **(C)** CCL3, CCL4, CXCL8, IL-1*β*, NETs and TNF were elevated, phagocytic and chemotactic activities were decreased. BioRender.com was used to create this.

The PRR signaling pathways and inflammatory response mechanisms induced by various strains of MVs also differ (Vanaja et al., 2016). Toll-like receptor 4 (TLR4) on the membrane surface of airway epithelial cells as PRRs may bind to MVs of bacteria such as *P. aeruginosa*, *E. coli*, *S. typhimurium*, and *Acinetobacter baumannii* (Zhao et al., 2013), stimulating the production of MyD88, IL-1*β*, NF-*κ*B, and so inducing inflammation. MVs can interact with a variety innate immune cell, the most well-studied of which is macrophages. MVs interact with macrophages, causing pro- or anti-inflammatory responses (Gilmore et al., 2021) (see Figure 3). The uptake and immunological response of the monocyte–macrophage system are directly linked to the distribution of MVs in diverse bodily organs (Jang et al., 2015). In macrophages, *Neisseria meningitides* MVs increase the expression of HLA-DR, CD80, CD86, and ICAM-1. Experiments in mice (Schertzer and Whiteley, 2013) revealed that *Salmonella* and *P. gingivalis* MVs stimulated macrophages and encouraged the creation of pro-inflammatory mediators. Furthermore, *P. gingivalis* MVs inhibited macrophage surface CD14 expression and accelerated CD14 degradation, resulting in reduced macrophage response to LPS (Winter et al., 2014) and a concomitant lack of response to antigen-stimulated secondary infection, an immunosuppressive state that worsens the inflammatory response (Cecil et al., 2017). PorB, a pore protein found in Gonococcal MVs, can shuttle into the mitochondria of host macrophages (Deo et al., 2018).

#### Modulatory effects on intrinsic immunity

By attaching to PRRs on the surface of immune cells via PAMPs, MVs can innate immune responses. MVs also activate intrinsic host sensing mechanisms (e.g., the inflammatory vesicle pathway) (Pathirana and Kaparakis-Liaskos, 2016), and in particular, MVs increase caspase-11-mediated atypical inflammatory vesicle responses via MVs bound LPS (Chen et al., 2018). Once internalized by host epithelial cells, MVs produced by bacteria such as *Helicobacter pylori*, *Neisseria gonorrhoeae*, and *P. aeruginosa* are recognized by the cytoplasmic immune receptor NOD1, making them susceptible to NOD1-dependent adaptive immune responses (Kaparakis et al., 2010; Irving et al., 2014).

#### Regulation of dendritic cells

MVs decrease DC responsiveness to cytokines associated with secondary bacterial antigens while inducing dendritic cell (DC) maturation and promoting antigen presentation (Kaparakis-Liaskos and Ferrero, 2015; Schetters et al., 2019) (see Figure 3). *Neisseria meningitides* MVs can aid in the development of DCs and encourage the synthesis of inflammatory mediators, including *Salmonella* spp. MVs cause DCs to express CD80, CD86, and MHC class II molecules. C-type lectin receptor (CLR) can facilitate DC attachment to integrin CD11c during antigen presentation, triggering the organism’s intrinsic and acquired immunity (Demento et al., 2011). *Salmonella*-derived MVs drive dendritic cells to express CD86 and TNF-α and IL-12 in the major histocompatibility complex (Imayoshi et al., 2011). *Salmonella typhimurium* MVs aid in the growth and maturation of BMDCs, which are inflammatory cytokine-producing cells generated from mouse bone marrow (Alaniz et al., 2007). By attaching to the host factor bactericidal permeability-increasing protein (BPI), *N. meningitides* MVs can facilitate their transport and internalization to dendritic cells (Schultz et al., 2007). A NOD1-dependent mechanism that polarizes T cells toward inflammatory Th2/Th17 responses is used by *V*. *cholera* O395 MVs to control epithelial pro-inflammatory responses and activate dendritic cells (Chatterjee and Chaudhuri, 2013).

#### Regulation of neutrophils

MVs stimulates the expression of PMN (polymorphonuclear neutrophil) mRNA and produces pro-inflammatory cytokines and chemokines, such as TNF-*α*, IL-1*β*, IL-8, macrophage inflammatory protein 1*α* (MIP-1*α*), and MIP-1*β*. *Neisseria meningitides* MVs provoke an inflammatory reaction and PMN stimulation (Lapinet et al., 2000). Cytotoxic necrosis factor type 1 (CNF1), which hinders PMN chemotaxis and phagocytosis, is present in both non-pathogenic and uropathogenic *E. coli* MVs (Davis et al., 2006). In the lungs of mice, neutrophil migration was accelerated by *E. coli* MVs (Lee et al., 2018). MVs work with oral epithelial PRRs to activate cells and release inflammatory factors, such as IL-6, IL-8, MCP-1. These factors then entice neutrophils to infiltrate periodontal tissues, where they can eventually lead to the degeneration of periodontal tissues and the onset of chronic periodontitis (Cecil et al., 2019).

#### Effects on adaptive immunity

MVs *in vivo* testing result in beneficial cellular and humoral immune reactions (Prados-Rosales et al., 2011). The MVs of *P. gingivalis* diminish the expression of HLA-DR and MHC-II molecules in endothelial cells, consequently limiting antigen presentation and adaptive immunological responses in peripheral blood and inhibiting the influence on capillary formation (Cecil et al., 2019).

#### Effect on antigen presentation

MVs contain a large number of PAMPs that stimulate the immune system, and APCs are necessary for the formation of adaptive immunological responses. APCs are activated by PAMPs found in MVs that bind with the appropriate pattern recognition receptors on APCs (Bachmann and Jennings, 2010). MVs have the necessary size to allow their entrance into lymphatic channels and efficient APCs absorption. Antigens delivered by MVs are presented by APCs (Liu et al., 2018; Tan et al., 2018). MVs with alloantigen on their surface, as revealed by Kuipers et al. (2015), handled antigens well and triggered cellular immunological response, which the physical binding of PAMPs may have enhanced. Inhibit antigen presentation and class antigen expression (Bomberger et al., 2014).

#### Effects on T and B cells

The suppression of T-cell immunity by MVs is manifested by the inhibition of cytokine secretion and T-cell proliferation. The mechanism of T cell suppression by *H. pylori* MVs was discovered to be stimulating monocytes to overexpress COX-2, IL-6, and IL-10 (Hock et al., 2017), inhibiting CD4+ T cells proliferation and inducing T cells apoptosis, resulting in immune modulation (Winter et al., 2014). *Bacteroides fragilis* MV-derived polysaccharides are sensed by intestinal dendritic cells via TLR2, promoting IL-10 expression, which then improves the function of regulatory T cells and suppresses intestinal inflammation (Chu et al., 2016). MVs activate immune tolerant cells from the intestine, which regulate other tissue-resident T cells and inflammation (Winter et al., 2014).

B-cell activation and antibody production are aided by MVs. MVs carry parental bacteria’s virulence molecules, giving them immunomodulatory capabilities to reduce B-cell resistance *in vivo* (Kuipers et al., 2018). MVs from *Moraxella catarrhalis* and *H. influenzae* could promote B cell proliferation (Deknuydt et al., 2014) and significantly increase IgG and IgM secretion from B cells by cross-linking with immunoglobulin, IgD BCR, or binding to TLR9 receptor. The IgD-binding (MID) protein on *Moraxella* MVs is required for B-cell receptor uptake (Perez Vidakovics et al., 2010), induction of IL-6 production, and increased IgM production following MVs B-cell uptake.

### Toxic effects of MVs

By delivering virulence factors and antibiotic-resistance genes, MVs can aid in the spread of bacterial pathogenicity. When MVs interact with host cells, they release active toxins and other virulence factors, leading to virulence (Dineshkumar et al., 2020). The enterohemorrhagic *E. coli* (EHEC) MVs serve as transporters for virulence factors and delivery tools for the virulence cargoes into the host cells (Bielaszewska et al., 2018). MVs also perform a variety of functions that aid in bacterial infection of host cells (Schwechheimer and Kuehn, 2015). *E. coli* secretes MVs of varying sizes to cause varying levels of DNA damage in intestinal epithelial Caco-2 cells (Ling et al., 2019). TLR responses in macrophages are activated by *E. coli* MVs, leading to the production of IFN- coli MVs activate TLR r1, which induces antiviral responses in cells (Gilmore et al., 2021). *H. pylori* MVs may increase virulence, and *Helicobacter pylori* MVs carrying CagA affect cellular junctions and associated regulatory proteins (Hatakeyama and Higashi, 2005). MVs from *A. baumannii* can deliver the virulence effector protein AbOmpA to cells, increasing cytotoxic cell death (Jin et al., 2011). The MVs of *P. aeruginosa* contain a number of virulence factors. These virulence factors enter the host cell’s cytoplasm via actin and are rapidly distributed to specific sites that affect host cell metabolism and function (Bomberger et al., 2009). *Pseudomonas aeruginosa* MVs can secrete a virulence factor, Cif, into the airways to suppress the immune response (Zhao et al., 2013). MVs secreted by periodontal pathogens contain molecules that can disrupt intercellular junctions and destroy the oral epithelial barrier (Nakao et al., 2014), as well as toxic molecules like lipooligosaccharides and outer membrane proteins (Tanabe et al., 2008). In the GI tract, MVs penetrate the mucus layer into the gastrointestinal epithelium and accelerate disease progression by secreting toxins (Canas et al., 2018).

#### Promoting the role of apoptosis

MVs can enter the cytoplasm of macrophages, leading to cellular inflammation and even death (Cecil et al., 2017; Fleetwood et al., 2017; Deo et al., 2018). Soluble VacA in *H. pylori* outer membrane vesicles induces apoptosis in gastric epithelial cells via a mitochondria-dependent pathway and generates reactive oxygen species (ROS), which induce cells to form autophagic vesicles, resulting in apoptosis (Yahiro et al., 2015). Macrophage death typically occurs 20 h after MVs treatment, whereas macrophages die rapidly within 35–48 h (Deo et al., 2020). The MVs of Gonococcal also trigger caspase-11-dependent apoptosis. Purified MV-mediated AMPK activation acts as an early warning system, initiating autophagy prior to bacterial invasion (Losier and Russell, 2019). MVs deliver LPS to the cytoplasm, stimulate IL-1*β* expression through caspase-11 dependence, and cause inflammation-induced programmed cellular scorching (Vanaja et al., 2016).

### MVs deliver sRNA cargo to host cells

By attaching to the mRNA of their host species to control gene expression, Small RNA (sRNA) in bacteria function similarly to their eukaryotic counterparts, micro-RNA binding to the mRNA of Diallo and Provost (2020). It has been demonstrated that *P. aeruginosa*’s immunomodulatory sRNA 52,320 enters human airway epithelial cells via MVs, reducing MVs-induced cytokine secretion and neutrophil infiltration *in vivo* and downregulating the synthesis of inflammatory factors *in vitro* (Koeppen et al., 2016). In addition, sRNA species were found in MVs from periodontal pathogens *P. gingivalis*, *Actinobacillus actinomycetemcomitans*, and dental dense spirochetes, which could inhibit the secretion of IL-5, IL-13, and IL-15 by Jurkat T cell (Choi et al., 2015). This provides further evidence that *H. pylori* employs MVs to reduce host immunity and boost pathogen survival by exploiting variations in immunomodulatory sRNA species (Zhang et al., 2020).

Additionally, the sRNA generated by MVs increases TNF-*α* production by activating the NF-*κ*B signaling pathway in macrophages, thereby promoting the secretion of pro-inflammatory cytokines in the brain (Choi et al., 2017; Ha et al., 2020). Bacilli actinomycetes are periodontal disease pathogens that can cross the blood–brain barrier. *Listeria monocytogenes* produces sRNA rli32 in MVs, which promotes intracellular development by stimulating the production of IFN-*β*in bone marrow-derived macrophages (Frantz et al., 2019). had their suppressed by Two sRNAs (sR-2509025 and sR-989262) released by *H. pylori* in MVs inhibited the secretion of IL-8 by human gastric cancer cells grown in the presence of LPS (Zhang et al., 2020). Sal-1 was identified as a sRNA that let *Salmonella* survive inside infected cells (Gu et al., 2017). In *V. cholerae* A1552, O1wasdiscovered a new sRNA gene called *vrrA*. To our knowledge, *vrrA* is the first sRNA to regulate MVs production, and VrrA can boost MVs production. In addition, the *vrrA* mutant was five times as effective as the wild type at colonizing the gut of newborn mice (Song et al., 2008).

### MVs exhibit different roles in different parts of the host

#### Intestinal tract

MVs avoid the degradation of encapsulated contents and enable for long-distance transport *in vivo* (Schertzer and Whiteley, 2013), and MVs can transport large amounts of nucleic acids to their functionally relevant destination. The findings indicated that MVs could enter the intestinal barrier and cause systemic inflammation via epithelial bypass (Jones et al., 2020). Pathogenic the LT intolerable toxin may be packaged by *E. coli* as MVs, while the CT cholera toxin can be packaged by *V. cholerae* (Chatterjee and Chaudhuri, 2011). These toxins cause watery diarrhea by altering intracellular cAMP levels, which causes cells to discharge more water into the colon (Kopic and Geibel, 2010). *Helicobacter pylori* MVs can cause micronuclei formation, altered iron metabolism and oxidative stress in human gastric epithelial cells (Chitcholtan et al., 2008). In a sulfate esterase-dependent way, MVs produced by commensal gut bacteria B can across the intestinal epithelial barrier of colitis-prone mice, causing intestinal inflammation (Hickey et al., 2015). By their virulence factors, *E. coli* MVs stimulate the host immune system, inducing sepsis by prompting host macrophages to generate IL-6 and TNF-*α* (Kim et al., 2018). Exposure of macrophage to MVs from *Legionella pneumophila*, *Neisseria gonorrhoeae*, uropathogenic *E. coli*, and *P. aeruginosa* promotes mitochondrial apoptosis and NLRP3 inflammatory vesicle activation (Deo et al., 2020). MVs produced by the intracellular pathogen, *L. pneumophila*, causing production of IL-6, IL-8, IFN-*γ*, MCP-1, and G-CSF, etc. *Helicobacter pylori* MVs increase the release of anti-inflammatory IL-10 from human peripheral blood mononuclear cells (PBMCs) (Fleetwood et al., 2017) while decreasing cytokine reactions, a result similarly found when *P. gingivalis* MVs interact with macrophages.

#### Oral tissues and promotion of biofilm formation

*Porphyromonas gingivalis* secrets MVs containing gingival pain circulate distant organs (Qing et al., 2020). MVs isolated from *P. gingivalis* can inhibit TLR4 and mTOR signaling, rendering monocytes nonresponsive to live bacteria. In addition, these MVs have been demonstrated to downregulate the expression of anti-atherogenic endothelial-type nitric oxide synthase (eNOS), while upregulating the expression of inducible nitric oxide synthase (iNOS) (Jia et al., 2015). Both foam cell production and platelet aggregation were induced by this MVs, both of which can contribute to the development of cardiovascular disease (Qi et al., 2003).

The addition of the MVs fraction of strain TK1402, which has a high biofilm-forming capability relative to other strains and is highly related with the generation of MVs, improves biofilm formation in of *H. pylori* strain (Yonezawa et al., 2009). *Porphyromonas gingivalis* MVs have been shown to increase the aggregation and adhesion of several other oral microbes in dental plaque biofilms, demonstrating the potential for MVs from one organism to promote the adhesion of another in biofilms (Kamaguchi et al., 2003). The attachment of whole-cell hyphae to epithelial cells was facilitated by the release of MVs carrying *P. gingivalis* (Inagaki et al., 2006). Research suggested that *P. gingivalis* MVs play a role in shaping the bacteria profile of periodontal plaque.

#### Respiratory tract

*Pseudomonas aeruginosa* vesicles stimulate the non-phagocytic NOD1 response as well as the production IL-8 synthesis in lung epithelial cells. MVs from other respiratory infections have been shown to trigger cytokine production. *Klebsiella pneumoniae* MVs cause epithelial cells to produce pro-inflammatory IL-8 and IL-1*β* after intratracheal injection, mimicking the inflammatory response in a neutropenic animal model of the disease (Bauman and Kuehn, 2006). *Staphylococcus aureus* MVs can migrate from the oropharynx to the skeletal system and enter osteoblasts and synovial cells via internalization (Chen et al., 2018), including cytogenic factors, such as GM-CSF and IL-6, to cause inflammation and bone tissues destruction. MVs produced by the intracellular pathogen *L. pneumophila*, causing production of IL-6, IL-8, IFN-*γ*, MCP-1, and G-CSF, etc.

### Beneficial effects of MVs

While many researches on MVs and their interactions with host cells has focused on pathogenic species, new publications have described the impact of MVs from commensal bacteria on host cells, especially those in the intestinal tract. Beneficial effects of probiotics on gut function include the prevention or reduction of symptoms of certain diseases. This is accomplished in part through probiotics’ ability to modulate host immune response; however, probiotics can also achieve this goal by competitively rejecting pathogenic bacteria and strengthening the intestinal epithelial cell barrier (Plaza-Diaz et al., 2017). While research into the precise methods by which probiotics exert their effects continues, it is becoming increasingly clear that MVs mediate communication between bacteria and host cells. Since mucus in the gut prevents bacteria from coming into direct contact with intestinal epithelial cells, it has been unclear how probiotics affect the host. Mucus-penetrating MVs are being increasingly acknowledge as an essential link in the communication network between bacteria and their hosts (Caruana and Walper, 2020). Pro-inflammatory cytokines are down-regulated in human intestinal epithelial cells in response to *B. fragilis* MVs, while anti-inflammatory cytokines (IL-4 and IL-10) are up-regulated (Ahmadi Badi et al., 2019). Nissle 1917, an *E. coli* probiotic, has been proven to have a protective effect against colitis via its MVs (Fábrega et al., 2017). Two proteins, p40 and p75, have been identified from cultures of *Lactobacillus casei* that have anti-apoptotic and cytoprotective activities, providing support to this notion (Dominguez Rubio et al., 2017). Because probiotics have no negative effects on the host, the use of these bacteria and/or their mv may pave the way for the creation of new cancer treatments that do not have the bad side effects of present chemotherapeutic drugs (Sharaf et al., 2018).

### Host cellular EVs act on bacteria

We discussed several of the impacts of MVs on host cells earlier in the text, and now we would like to briefly explore host EVs’ effects on bacteria. *Mycobacterium tuberculosis* infection alters exosome composition, and it was found that exosomes secreted by macrophages infected with *M. tuberculosis* contained mycobacterial lipid components that induced a proinflammatory response in uninfected macrophages (Giri and Schorey, 2008). Exosomes generated from *Salmonella*-infected macrophages contain pro-inflammatory factors that promote monocyte TNF-*α* expression and stimulate the activation of uninfected macrophages (Bhatnagar et al., 2007). Exosomes from *E.coli*-infected macrophage were found as a result of the same pro-inflammatory outcome (Imamiya et al., 2023). Huang et al. (2010) found that miR-155 was also significantly up-regulated in exosomes from Hp-infected macrophages, and that exosomes carrying miR-155 were taken up and internalized by macrophages to regulate the expression of various pro-inflammatory mediators and inflammation-associated proteins in macrophages. Exosomes have a complex function in the regulation of inflammatory responses, and whether this is due to different targets of exosome action or activation of signaling pathways has to be investigated further (Cao et al., 2019).

## MVs clinical applications

MVs have emerged as candidates with strong clinical potential, including application in cancer therapy, vaccine development, and antimicrobial therapy, based on their distinct features for cost effectiveness in production, stability to transport and storage, ease of modification, and proven immunomodulatory properties. In this section, we mainly focus on recent advances in the fields of vaccines, medicines, adjuvants, and drug carriers on MVs.

### Applications in vaccines

**Figure 4:**
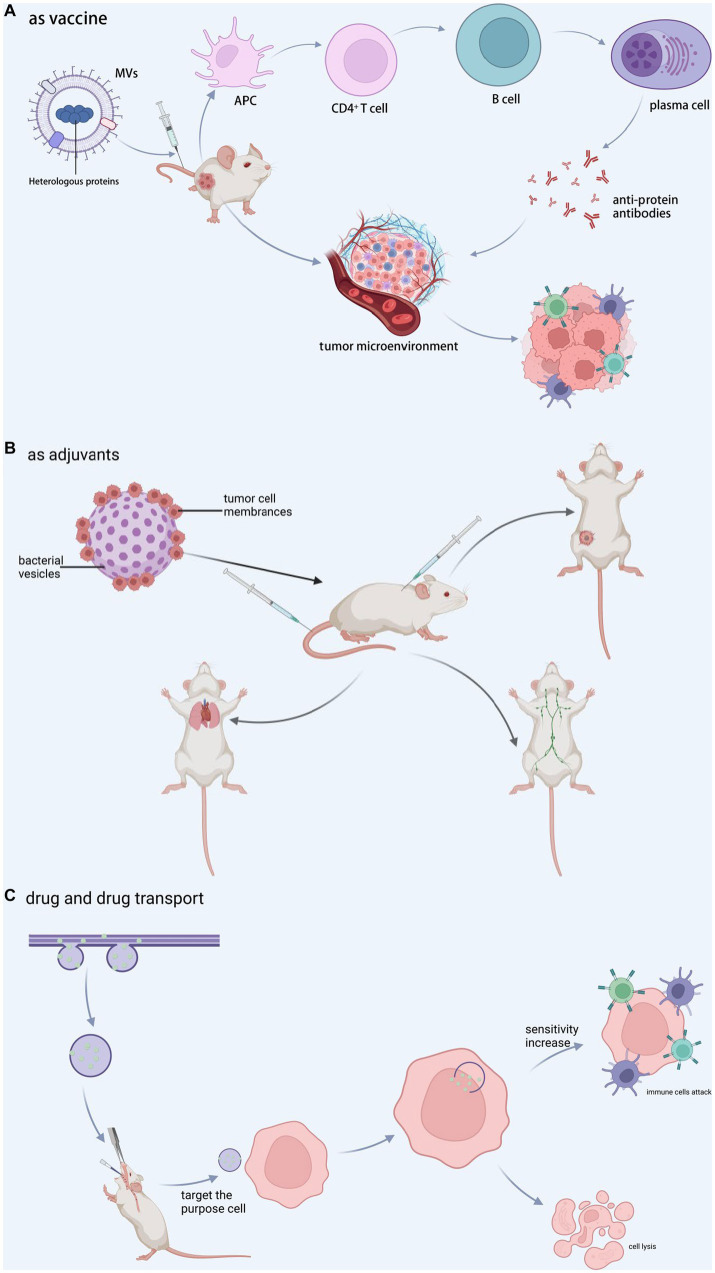
MVs in clinical applications. **(A)** Vaccine-associated proteins are encapsulated during vesicle formation, and the complex is administered into tumor-bearing animals, resulting in a decrease in tumor cells and an increase in CTL cells via immunomodulation or direct interaction with tumor tissue. **(B)** Tumor cell membranes and bacterial vesicles are combined to generate new vesicles, which are then used as adjuvants in customized immunotherapy, inguinal lymph node activation, lung metastasis inhibition, and recurrence inhibition. **(C)** The medication penetrates during vesicle formation and afterwards targets the target cells, increasing cell sensitivity and encouraging target cell attack and cell destruction by immune cells. BioRender.com was used to create this.

Traditional vaccines are intended to protect humans from pathogenic infections that cause diseases. Vaccines can elicit a strong and long-lasting pathogen-specific immune response by activating both innate and adaptive immunity by mimicking a pathogen without causing the associated disease. MVs are a high-quality candidate antigen in the vaccine development chain because it contains a large amount of highly immunogenic material and cannot replicate. The most well-known is the group B meningococcal vaccine, which was developed and manufactured to treat epidemic meningomyelitis caused by *N. meningitides*. MVs have been widely used in meningococcal vaccines against group B (Committee on Infectious Diseases, 2016; Masforrol et al., 2017). Researchers typically treated MVs with decontaminants (e.g., sodium deoxycholate, etc.) in the early stages of MVs vaccine development to remove a large amount of LPS present (Christodoulides and Heckels, 2017). MVs vaccines gained sufficient safety as a result. The ability of MVs to be embellished to express a scope of exogenous epitopes and the simplicity and cost-effectiveness of large-scale production make it attractive as a novel vaccine technology (Rappazzo et al., 2016). By incorporating heterologous proteins with specifically presenting antigens into MVs, the antigen can maintain its natural conformation while targeting specific immune responses (see Figure 4A). MVs can be genetically bedecked to express various exogenous epitopes. It is simple and inextensive to manufacture on a large scale, making it appealing as a novel vaccine technology (van de Waterbeemd et al., 2013). However, various critical factors, such as protein loading efficiency, antigen immunogenicity, immune response strength, and antigen delivery system targeting, should be evaluated. Exon structural domain matrix protein 2 by *E. coli* genetically engineered MVs, and vaccination with such MVs as a vaccine protects animals against the influenza A (H1N1) virus (Rappazzo et al., 2016). Pertussis proteoliposomes or MVs (dOMVBP) were created from inactivated *Bacillus pertussis* strain 165 whole cells. Pertussis toxin, bacterial hairs, and regio-stats are all present the pertussis proteoliposome MVs (dOMVBP), which was created using inactivated *B. pertussis* strain 165 whole cells. In intracerebral and intranasal challenge models, the dOMVBP vaccine is highly protective against WHO strain 18,323 (Asensio et al., 2011). In addition, intranasal vaccination with MVs may open a rapid vaccine approach (Pritsch et al., 2021).

### MVs as adjuvants

Adjuvants form depots, increase antigen uptake and presentation, deliver antigens to lymph nodes, and directly activate innate immunity (Awate et al., 2013) (see Figure 4B). As a result, adjuvants may reduce the antigen dose and the doses required to achieve prophylactic and therapeutic effects, lowering vaccine costs and making vaccination more convenient. The size of MVs may facilitate their passage through lymphatic drainage or their phagocytosis and subsequent carriage by APCs (Gerritzen et al., 2017). The LPS on the surface of MVs is a typical TLR4 activator, which is one of the reasons why MVs could be developed as an adjuvant (Kaparakis-Liaskos and Ferrero, 2015). The toxic moiety of LPS is a glycolipid known as lipid A, which is made up of a bis-phosphorylated diglucosamine backbone with up to seven acyl chains linked by ester and amide connections. This modified LPS retains the adjuvant activity while being much less toxic as a pyrogen to humans. The antigen-loaded MVs can be seen not only on the surface but also in the lumen of MVs (van der Pol et al., 2015; Gerritzen et al., 2017). The benefit of such a modification is the protection of antigens within the MVs lumen. These features above make it possible to develop MVs as an adjuvant.

### MVs as medicines

MVs is a promising candidate for therapeutic agents that can be delivered to targeted sites *in vivo*. Selective targeting of tumor cells using homologous ligands for tumor cell receptors may enable MV-based therapies with more specificity and fewer untoward effects than conventional unselective chemotherapy. MVs can be recognized, endocytosed, and digested by GI cells without the need for targeting ligands, which holds promise for treating GI tract tumors (Bielaszewska et al., 2017). In a research targeting HER2 receptors over-expressed on tumor cell membranes, *E. coli* MVs in mice with high specificity and weaken tumor load was designed to carry anti-tumor siRNAs and express HER2 ligands to target tumors (Gujrati et al., 2014). Under near-infrared light irradiation at 808 nm, a single low-dose injection of *S. typhimurium* MVs resulted in extravasation of erythrocytes from the tumor, causing the tumor to appear black, and adequate clearance of breast and colon cancers (Zhuang et al., 2021). MVs separated from transgenic *E. coli* DH5*α* over-expressing the tumor-derived protein “basic fibroblast growth factor” (BFGF), which gathered in functional BFGF protein, which stimulated anti-BFGF antibody production in mice. Repeated immunization with BFGF-enriched MVs inhibited tumor angiogenesis, overcame the immune restrain tumor microenvironment, and induced tumor-specific cytotoxic T lymphocytes (Huang et al., 2020a) (see Figure 4C).

### As drug carrier

MVs can transport drugs to specific sites and then release them to improve disease treatment. In a mouse model of intestinal *E. coli* infection, *A. baumannii* MVs encapsulated with levofloxacin can effectively invade *E. coli*, *P. aeruginosa*, and *A. baumannii* and cause effective killing of *E. coli*, as well as produce good therapeutic effects (Huang et al., 2020b). Shi et al. (2020) enriched *E. coli* MVs at the colon site. When the MVs rupture, the drugs they contain can be released at the site of colon cancer lesions. MVs can transport antitumor drugs such as tegafur (Chen et al., 2020), doxorubicin (Kuerban et al., 2020), paclitaxel, small inhibitory RNAs (Guo et al., 2021), and others. Antibiotics encapsulated in *S. aureus* MVs with active targeting were used to transport drugs into cells and destroy intracellular *S. aureus* (Gao et al., 2019). MVs encapsulated drug, as a novel nanomedicine, can activate host immune response regulation to tumor and deliver drug micelle chemotherapy to tumor, making cancer cells sensitive to CTL, killing, and inhibiting metastasis (Chen et al., 2020) (see Figure 4C).

## Summary and outlook

Over the past decade, our understanding of MVs function has grown rapidly, as has our comprehending of the mechanisms of MVs biogenesis, but numerous pivotal questions about their production and function remain no replied.

Current genetic engineering of vesicles is focused on genetic modification of plasmid transformation, allowing heterologous outer membrane and periplasmic protein expression in *E. coli* loaded into secreted expression of alternative vaccine antigens as vector therapeutic cargoes, or nano vector targeting of biologic cargoes to specific target cells to make them attractive for new therapeutic technologies, all of which are “additive.” MVs from TrxA-deficient *A. baumannii* clinical isolate Ci79 caused more J774 macrophage-like cell death than wild type MVs. This DtrxA MV-mediated cell death was vanished when cells were hatched with protease K treated MVs (Shrihari et al., 2022). “Subtraction” may become a new way. To reduce the virulence of the MVs by genetic engineering to attenuate or inhibit the virulent proteins of the bacteria, thereby reducing the virulence of the proteins carried on the MVs while still allowing the MVs to invade the host cell and deliver effective substances to intervene and treat the host cell. Furthermore, MVs produced by *P. gingivalis* effectively protect cells from chlorhexidine and have a degradative enzymatic activity to neutralize the killing ability of human serum. MVs delivered by accelerate wound closure by injection would closure PD-L1-expressing myeloid cells to the injured parts (Alpdundar Bulut et al., 2020). The ability of MVs to reach other parts of the host organism away from the bacterium, and the close association of MVs surface markers with the bacteria of origin, may be a key feature for further studies to further understand its biological function by diagnosing the cause and mechanism of disease occurrence through the detection of markers in distant organs. Although pure MVs have been shown to eliminate tumors by triggering the upregulation of interferon *γ* (Kim et al., 2017), a function used for cancer therapy, the construction of a functional MVs platform capable rapidly displaying multiple tumor antigens is critical for the development of personalized tumor vaccines. Future regulation of MVs biogenesis and composition will contribute to a better understanding of MVs pathogenic mechanisms and disease states.

In conclusion, while we do not yet fully comprehend MVs, this does not preclude further investigation of its practical applications. In the field of antitumor vaccines, MVs offers significant advantages and promises. We have reason to believe that by continuing to investigate the physiological properties of MVs and the immune mechanisms they elicit, we will be able to fully exploit their potential applications and eventually form a new MV-based disease prevention and control platform that will significantly contribute to the prevention and treatment of cancer and inflammatory diseases.

## Author contributions

MX and GL wrote the manuscript. GL and HY revised the manuscript and provided guidance on the structure and content. All authors contributed to the article and approved the submitted version.

## Funding

This research was funded by the National Natural Science Foundation of China (grant no. 82260186), and Natural Science Foundation of Yunnan Province (grant nos. 202002AA100007, CXTD201904, Yun ren she tong (2019) 206, 202101AT070150).

## Conflict of interest

The authors declare that the research was conducted in the absence of any commercial or financial relationships that could be construed as a potential conflict of interest.

## Publisher’s note

All claims expressed in this article are solely those of the authors and do not necessarily represent those of their affiliated organizations, or those of the publisher, the editors and the reviewers. Any product that may be evaluated in this article, or claim that may be made by its manufacturer, is not guaranteed or endorsed by the publisher.
